# Physiological and Psychological Predictors of Short-Term Disability in Workers with a History of Low Back Pain: A Longitudinal Study

**DOI:** 10.1371/journal.pone.0165478

**Published:** 2016-10-26

**Authors:** Jean-Daniel Dubois, Vincent Cantin, Mathieu Piché, Martin Descarreaux

**Affiliations:** 1 Département de psychologie, Université du Québec à Trois-Rivières, Trois-Rivières, Québec, Canada; 2 Département des sciences de l’activité physique, Université du Québec à Trois-Rivières, Trois-Rivières, Québec, Canada; 3 Département de chiropratique, Université du Québec à Trois-Rivières, Trois-Rivières, Québec, Canada; Universita degli Studi di Perugia, ITALY

## Abstract

Despite an elusive pathophysiology, common characteristics are often observed in individuals with chronic low back pain (LBP). These include psychological symptoms, altered pain perception, altered pain modulation and altered muscle activation. These factors have been explored as possible determinants of disability, either separately or in cross-sectional studies, but were never assessed in a single longitudinal study. Therefore, the objective was to determine the relative contribution of psychological and neurophysiological factors to future disability in individuals with past LBP. The study included two experimental sessions (baseline and six months later) to assess cutaneous heat pain and pain tolerance thresholds, pain inhibition, as well as trunk muscle activation. Both sessions included the completion of validated questionnaires to determine clinical pain, disability, pain catastrophizing, fear-avoidance beliefs and pain vigilance. One hundred workers with a history of LBP and 19 healthy individuals took part in the first experimental session. The second experimental session was exclusively conducted on workers with a history of LBP (77/100). Correlation analyses between initial measures and disability at six months were conducted, and measures significantly associated with disability were used in multiple regression analyses. A first regression analysis showed that psychological symptoms contributed unique variance to future disability (R^2^ = 0.093, p = .009). To control for the fluctuating nature of LBP, a hierarchical regression was conducted while controlling for clinical pain at six months (R^2^ = 0.213, p < .001) where pain inhibition contributed unique variance in the second step of the regression (R^2^ change = 0.094, p = .005). These results indicate that pain inhibition processes may constitute potential targets for treatment to alleviate future disability in individuals with past or present LBP. Then again, the link between psychological symptoms and pain inhibition needs to be clarified as both of these factors are linked together and influence disability in their own way.

## Introduction

Of all musculoskeletal pain conditions, low back pain (LBP) is the most common, with an estimated worldwide 1-month prevalence of 23.2% [1] and a lifetime prevalence of up to 84% [2]. Such a high prevalence, and the numerous therapeutic interventions used for nonspecific LBP greatly increase the economic costs and burden of this condition on society [3, 4]. Since they always live with doubts as to when the next episode will strike [5, 6], many individuals with LBP report that their activities are limited and that they consciously make efforts to avoid pain recurrences when they are pain-free, or pain exacerbations when their pain is ongoing [7]. Most of these individuals still work, but with a decreased productivity [8, 9]. Moreover, flare-ups are characterized by increased pain causing additional activity limitations [7]. These recurrences of acute pain have been shown to mask the contribution of key variables in the prediction of disability in individuals with LBP [10]. Therefore, identifying factors that contribute to disability regardless of these fluctuating pain levels is critical to increase performance and productivity in the workplace.

Despite its high prevalence, nonspecific LBP and its underlying pathophysiology remains elusive. Even so, previous studies have noted that individuals with LBP often exhibit psychological distress, including increased pain catastrophizing [11], pain-related fear [12], anxiety [13], hypervigilance to pain [14] and avoidance behaviors [15]. Encompassing most of these factors, the fear-avoidance model of musculoskeletal pain [16] is now considered one of the most comprehensive model to understand the transition from acute to chronic pain [17]. As such, many of the psychological factors included in the fear-avoidance model have been identified as partially responsible for the development of short and long term disability in individuals with LBP [18]. Recently however, some authors have proposed that the fear-avoidance model of musculoskeletal pain could be reframed in order to include pain-related physiological processes [19]. This is consistent with numerous studies showing that neurophysiological alterations are frequent in individuals with LBP. These alterations include changes in neuromuscular activation of trunk muscles [20, 21] as well as hyperalgesia, localized to the lower back [22, 23] or widespread, which also affects other body areas [22–24]. Finally, some authors suggest that individuals with LBP may present pathological pain mechanisms such as altered pain inhibition processes [25] that are also reported in individuals with other chronic pain conditions [26].

In individuals with LBP, reduced pain thresholds [25], psychological factors and neuromuscular adaptations [27] have all been linked to increased disability. However, these cross-sectional studies focused on punctual disability, and because low back pain is a fluctuating condition, in terms of both disability and painful episodes, the relative contribution of all aforementioned factors to future disability remains unknown.

Therefore, the main objective of this longitudinal study was to determine the contribution of psychological factors, neuromuscular adaptations, pain thresholds and tolerance, as well as pain inhibition processes to disability recorded six months later in working individuals with a history of LBP. The main hypothesis was that at least one of the aforementioned factors, or their combination with clinical pain levels observed at six months would contribute to future disability in workers with a history of LBP.

## Methods

### Ethics statement

All experimental procedures conformed to the standards set by the latest revision of the Declaration of Helsinki and were approved by the *Université du Québec à Trois-Rivières* Research Ethics Board. All participants gave their written informed consent, acknowledging their right to withdraw from the research project or individual experimental session without prejudice. All participants received a compensation of CA$25 for each experimental session they attended, during an 18-month longitudinal study. The current report presents data from the first 6 months of the study.

### Participants

#### Workers with a history of low back pain

One hundred workers with at least one previous episode of disabling LBP were included as part of the 18-months longitudinal study (54 men and 46 women). Seventy-seven (39 men and 38 women) of these workers participated in the second experimental session six months later (reasons given by participants for leaving the study are presented in Table 1). All participants were recruited either through advertisement in the local newspaper, through the university outpatient clinic or amongst university personnel through internal advertisement. To be included in the study, any given participant needed to be currently employed (not on sick-leave at the beginning of the study) and had to have modified its work-related activities because of LBP at least once in the previous three years (either in the form of modified work or sick-leave). Participants between 18 and 60 years old were included. Exclusion criteria included inflammatory arthritis, osteoporosis, neuromuscular disease, herniated disk, radiculopathy and any other chronic pain syndrome (including, but not limited to, fibromyalgia, irritable bowel syndrome and chronic tension-type headaches). Participants were assessed at the beginning of the study by an experienced clinician to confirm the non-specific nature of low back pain.

**Table 1:** Reasons given for discontinuing participation in the project.

Reason for discontinuing participation	n
Did not respond to further inquiries (phone/email)	14
Moved outside of the city	5
Not enough time	2
Illness	1
Excluded because of a previously undisclosed chronic condition	1

#### Workers with no previous history of low back pain

In order to obtain normative values for predictive factors (psychological, neuromuscular and neurophysiological) assessed in workers with a history of LBP, 19 healthy workers (controls) were also recruited to take part in the first experimental session. All inclusion criteria for these controls were the same as for workers with previous LBP except that they needed to be free from any pain in the lower back for a minimum of three years (no controls had ever experienced an episode of LBP in the past). The exclusion criteria were the same as those used for workers with previous LBP. A broad spectrum of individuals were selected and care was taken to match the mean age, height, weight and type of work to those observed in workers with past LBP.

### Procedure

**Fig 1:**
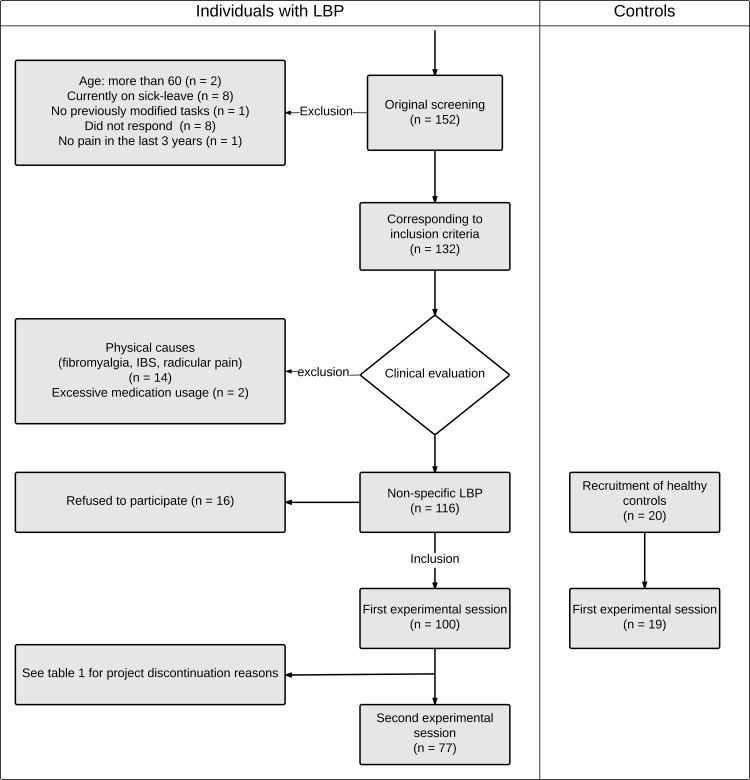
Progression of the study and number of participants taking part in each step.

Workers with a history of LBP were assessed at the beginning of the study and six months following baseline evaluation, whereas healthy controls were only assessed initially. The flowchart presented in Fig 1. conveys the unfolding of the study and groups taking part in specific sessions. Each experimental session was conducted at the “Laboratoire de Neuromécanique et de contrôle moteur de l’Université du Québec à Trois-Rivières” between February 2013 and October 2015. Experimental sessions included the completion of validated questionnaires as well as an interview to obtain information regarding past episodes and current low back pain (where applicable), medication and work leave. Along with questionnaires, each experimental session included 1) an assessment of pain thresholds and pain tolerance, 2) heterotopic noxious counter-stimulation (HNCS) to assess pain inhibition processes and 3) a dynamic task (flexion-extension of the trunk) combined with experimental LBP to evaluate neuromuscular adaptations and neuromuscular responses to experimental pain. Participants were contacted three months after the initial assessment to get information on pain episodes or ongoing pain since the initial visit. They were also asked to fill all questionnaires again.

### Questionnaires

Current clinical pain was assessed using a visual analog scale (VAS) at the beginning of each experimental session. Disability, fear-avoidance beliefs about physical activity and work, pain hypervigilance, pain catastrophizing and work satisfaction were respectively assessed using the Roland-Morris Disability Questionnaire (RMDQ) [28], the Fear-Avoidance Beliefs Questionnaire (FABQ), the Pain Vigilance and Awareness Questionnaire (PVAQ) [29], the Pain Catastrophizing Scale (PCS) [30], the Minnesota Satisfaction Questionnaire (MSQ) [31] and the STarT Back Tool [32]. All questionnaires were presented to participants in their French validated version [33–38].

#### Disability

The Roland-Morris Disability Questionnaire is a 24 items, simple to complete self-report assessing disability related to low back pain [28]. The score ranges from 0 (no disability) to 24 (maximum disability) and is calculated by adding the number of items checked [39].

#### Fear-avoidance beliefs

The Fear-Avoidance Beliefs Questionnaire (FABQ) consists of two subscales respectively pertaining to physical activity and work for a total of 16 items (5 items for physical activity and 11 for work) [15]. Each item is answered on a 7-point Likert scale from strongly disagree to strongly agree. The score for the physical activity subscale (FABQpa) is obtained by adding four of the five items (items 2, 3, 4 and 5) for a possible maximum of 24, whereas the score for the work subscale (FABQw) is obtained by adding items 6, 7, 9, 10, 11, 12 and 15 for a maximum of 42 [40].

#### Pain hypervigilance

The Pain Vigilance Awareness Questionnaire (PVAQ) is a 16-item measure of attention to pain that also assesses awareness and vigilance to pain [29]. Respondents are asked to consider their awareness to pain in the previous two weeks and indicate, on a scale of 0 (never) to 5 (always), how often they engage in such behavior [29].

#### Pain catastrophizing

The Pain Catastrophizing Scale (PCS) is a 13-item scale used to assess the presence of pain catastrophizing. The total score ranges from 0 to 52, with a higher score indicating increased level of pain catastrophizing [30].

#### Work satisfaction

The short-form Minnesota Satisfaction Questionnaire (MSQ) is a scale that measures intrinsic, extrinsic and general work satisfaction. For each of the 20 items, five responses can be given (very dissatisfied (1), dissatisfied (2), neither satisfied nor dissatisfied (3), satisfied (4) or very satisfied (5)) and the total score ranges from 20 to 100 [31].

#### Primary care back pain screening tool

The Subgroups for Targeted Treatment (STarT) Back Screening Tool (commonly referred to as the STarT Back) is comprised of nine questions. One of these questions is related to how bothersome was the individual’s back pain in the last two weeks (with possible answers ranging from not at all (0), slightly (0), moderately (0), very much (1) and extremely (1)). All other items are dichotomic, with either “Agree” or “Disagree” as possible answers [32].

### Experimental pain assessment

Experimental pain in the form of cutaneous heat was rated using a validated numerical rating scale (NRS) with verbal and numerical anchors for no pain (0), light pain (21), moderate pain (46), strong pain (75), and extreme pain (97) [41]. All cutaneous heat stimuli were administered using a computer-controlled Medoc TSA-II Neurosensory Analyzer for Sensory Testing and a 9 cm^2^ contact thermode (Model TSA-2001; MEDOC Advanced Medical Systems, Ramat Yishai, Israel). Heat pain thresholds and heat pain tolerance were assessed using the ascending method of limits. Baseline temperature of the contact thermode was set at 32°C and increased at a rate of 1°C per second. For heat pain thresholds, participants were instructed to respond by the word “now” when the sensation first became painful (at which point a button was pressed to record the temperature) and to press another button when they “could no longer tolerate pain”, the latter corresponding to pain tolerance threshold. This protocol was carried out on the skin over the lower back (midline between the L4 and L5 spinous processes) and on the volar surface of the forearm (middle point between the medial condyle of the humerus and the styloid process of the ulna). Three trials were performed for each (low back and forearm) stimulation site and the site of the thermode was alternated for each trial. Participants were asked to rate pain intensity immediately after they pressed the button to discontinue the stimulation (pain tolerance). Noxious temperatures used to evoke moderate pain (40/100) during the counter-stimulation protocol and the flexion-extension task were individually adjusted using the ascending method of limits (increments of 0.5°C) with temperature ranging from 42 to 50°C.

### Heterotopic noxious counter-stimulation (HNCS)

**Fig 2:**
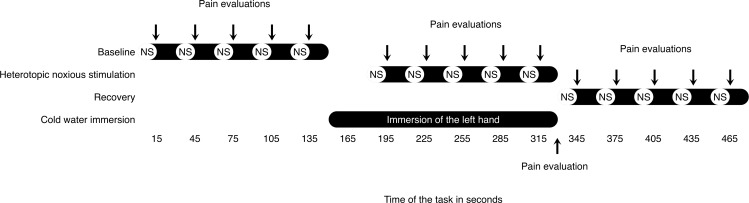
Experimental protocol of the heterotopic noxious counter-stimulation task.

This task lasted a total of 480 seconds and included a total of three 150-second blocks (baseline, HNCS and recovery) (see Fig 2). Each block comprised five 15-second noxious stimuli applied to the lower back each followed by a 15-second inter-stimulus interval. Once the first block (baseline) was completed, the left hand was immersed into circulating cold water (NESLAB RTE 211 Digital One, Thermo Scientific Co., Massachusetts, USA) 30 seconds prior to the beginning of the second block (HNCS). Temperature used for cold water immersion was determined at the beginning of the experimental session and adjusted individually to produce moderate pain (around 50/100). Once the last stimulation of the second block (noxious stimulation to the lower back) was completed, the hand was removed from cold water and wrapped in a dry towel. The last block (recovery) of stimuli was then administered. Participants were asked to rate pain intensity after each noxious stimulus (all cutaneous heat stimuli to the lower back as well as cold pain). For the duration of the experiment, participants were instructed to focus their attention on noxious stimulation to the lower back.

### Flexion-extension task

**Fig 3:**
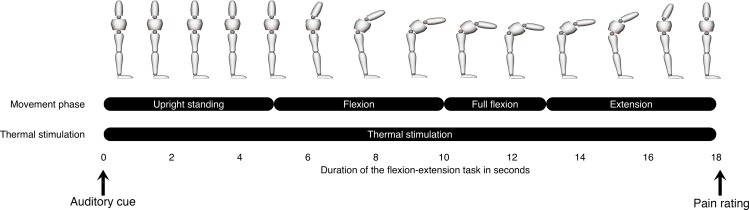
Experimental procedure illustrating the combination of a flexion-extension task with cutaneous heat applied to the lower back.

The task consisted of four movement phases: (1) upright standing, (2) trunk flexion to reach a fully-flexed state, (3) full flexion, and (4) trunk extension to return to the initial upright position. The task was explained and demonstrated before any experimental trial was undertaken. A metronome was used for movement pacing to ensure that upright standing, flexion and extension lasted five seconds, while full flexion was maintained for three seconds. A total of 15 flexion–extension cycles were performed and each experimental condition: (1) no stimulation, (2) innocuous cutaneous heat, and (3) noxious cutaneous heat randomly applied five times to the participant’s lower back. Noxious stimuli to the lower back were used as previous studies showed that increased disability in individuals with LBP was associated with decreased neuromuscular responses during experimental LBP [27, 42]. The beginning of the task was indicated by an auditory cue. Cutaneous heat was first applied and the flexion began five seconds after the desired thermode temperature was reached. After each flexion–extension cycle, participants were asked to rate pain intensity on a NRS and were given 1 min to rest before the next trial. The NRS was placed vertically in front of the participants in order to help them rate pain intensity induced by cutaneous heat. The flexion-extension cycle can be visualized on Fig 3.

### Neuromuscular adaptations

#### Electromyography

Surface electromyography (EMG) data were collected bilaterally using bar bipolar active surface electrodes applied over the lumbar erector spinae (LES) muscles at the L4–L5 and L2-L3 levels, approximately 3 cm from the mid-line (electrodes were applied in-line with muscle fiber direction). Electrode material was 99.9% Ag and the interelectrode distance was fixed at 10 mm. A ground electrode was placed on the left anterior superior iliac spine. Skin impedance was reduced by (1) shaving body hair, (2) gently abrading the skin with fine-grade sandpaper (Red Dot Trace Prep, 3M, St. Paul, MN, USA), and (3) wiping the skin with alcohol swabs. EMG activity was recorded using a single differential Delsys Surface EMG sensor with a common mode rejection ratio of 92 dB at 60 Hz, a noise level of 1.2 μV, a gain of 10 V/V ± 1%, an input impedance of 10^15^ Ω, a bandwidth of 20–450 ± 10% (Model DE2.1, Delsys Inc., Boston, MA, USA) and sampled at 1000 Hz with a 12-bit A/D converter (PCI 6024E, National Instruments, Austin, TX, USA). The EMG data were filtered digitally by a 10- to 450-Hz bandpass, zero-lag, fourth-order Butterworth filter. Data were collected using LabView (National Instruments, Austin, TX, USA) and processed by Matlab (R2007b MathWorks, Natick, MA, USA).

#### Kinematics

Kinematics data were collected by a motion analysis system (Optotrak Certus, Northern Digital, Waterloo, ON, Canada). Light-emitting diodes (LED) were positioned on the right side of the participants over six anatomical landmarks: (1) lateral condyle of the femur, (2) great trochanter, (3) anterior superior iliac spine (ASIS), (4) posterior superior iliac spine (PSIS), (5) L1 and (6) T11. Data were sampled at 100 Hz and low-pass filtered by a dual-pass, fourth-order Butterworth filter with a cut-off frequency of 5 Hz. Kinematic data assessment was synchronized with EMG assessment through a signal triggered by LabView.

### Data analysis

#### Pain and pain tolerance thresholds

Heat pain threshold and heat pain tolerance for each stimulation site were determined as the mean temperature from the three trials for each site (lower back and volar surface of the forearm).

#### HNCS protocol

All five pain ratings for each block of noxious heat stimulation were averaged. Pain inhibition was calculated by subtracting the mean pain during baseline from mean pain during HNCS (negative values represent better pain inhibition).

#### EMG and kinematics

Modulation of the surface EMG amplitude was calculated with a root mean square (RMS) value using a 250 ms window and a 150 ms overlap during each of the four movement phases for the L4-L5 and L2-L3 levels. Normalized RMS EMG values were obtained by dividing the mean RMS during each phase of movement by the RMS obtained during the extension phase of the reference trial. Since normalized RMS values of left and right erector spinae muscles (compared at each level) were not statistically different for each movement phase (t-tests, all p > 0.05), left and right RMS values were therefore averaged for all analyses [43]. For kinematics, two adjacent LED were used to create a vector and the angles between vectors served to quantify lumbar spine and pelvic motion. Lumbar spine motion was obtained by calculating the angle between the T11–L1 and ASIS-PSIS vectors. Pelvic motion was determined by the angle between the ASIS-PSIS and great trochanter-lateral condyle of the femur vectors. The total trunk flexion angle was obtained by adding the lumbar spine angle to the hip angle. RMS values of surface EMG activity were recorded throughout the flexion–extension task. RMS EMG (both levels) during full flexion (no stimulation condition), usually associated with the flexion relaxation phenomenon (FRP) [44], was used to determine the individuals’ baseline neuromuscular adaptations. Changes induced by noxious heat (experimental LBP) were determined for lumbar erector spinae (both levels) during the full flexion phase. In order to compute these changes, normalized RMS EMG obtained during the no stimulation condition was subtracted from normalized RMS EMG obtained during the noxious heat condition.

### Statistical analyses

T-tests for independent samples were used to compare the baseline characteristics (including age, height, weight, psychological symptoms, pain thresholds and tolerance thresholds) of all workers with a history of LBP (n = 100) to those of healthy controls (n = 19). Based on power calculation from our last two experiments (also conducted on individuals with past or present LBP) [27, 45] which showed high effect sizes for neuromuscular adaptations and psychological factors, 19 control participants was deemed to yield sufficient power (> 80%) when comparing baseline values with those from individuals with past LBP. To determine the effect of HNCS, a mixed-design analysis of variance (ANOVA) was conducted on cutaneous heat pain ratings before, during and after (within-subject) hand immersion in cold water for both groups (between-subject). The effects of experimental LBP on normalized RMS EMG and total trunk flexion angle were also assessed using mixed-design ANOVAs for the flexion, full flexion and extension phases (each electrodes placement for EMG) and for the whole task (total trunk flexion angle) with conditions (no stimulation, innocuous heat and noxious heat) as a within-subject factor, and groups (controls and patients) as a between-subject factor.

The following statistical analyses were only performed on individuals with past or present LBP as the main goal of the study was to determine the contribution of initially-assessed variables to future disability in these individuals. Principal component analyses (PCA) were conducted on questionnaire scores, pain thresholds and tolerance, and neuromuscular adaptations for data reduction. A first PCA was created using the fear-avoidance beliefs about physical activity, pain catastrophizing and pain hypervigilance questionnaires (all other psychological factors did not load on this component). Another PCA was created using pain thresholds and pain tolerance for both the lower back and the volar surface of the forearm. A third PCA was conducted with EMG activity at the L4-L5 and L2-L3 levels (baseline neuromuscular adaptations) and with changes in EMG activity (L4-L5 and L2-L3) induced by experimental LBP (neuromuscular responses to experimental pain). Each PCA met the criterion for extraction (communality all > 0.5) and sampling adequacy (KMO all p < 0.05) and individual variables loaded on a single component.

Correlation analyses were performed to examine the relation between the Roland-Morris disability scores obtained at six months and the three components yielded by PCAs, the total trunk flexion angle and pain inhibition (all variables at initial visit). Subsequently, a multiple regression analysis was conducted to explain disability at six months and variables significantly associated with disability were entered in the model (see correlation analyses above). A second hierarchical regression analysis was also conducted with clinical pain at six months entered in the first step of the regression. As stated earlier, persistent and recurrent LBP are characterized by fluctuations in pain and disability levels. Moreover, clinical pain levels can mask the contribution of otherwise meaningful factors to disability in individuals with LBP [10]. Once the variance from clinical pain was removed, the same variables previously associated with disability were entered in the second step of the regression. Residuals from the regression analysis were assessed using the Kolmogorov-Smirnov test in addition to visual inspection to assess normality of the distribution. Finally, collinearity was assessed by examining tolerance for each predictor entered in the multiple regression analysis. Statistical significance was set at p ≤ 0.05 for all analyses.

## Results

To ensure that participants who dropped out of the study were not different from those who returned six months later, all independent predictors were compared using t-tests for independent samples (all p > .192). Moreover, some participants who remained in the study had scarce missing values but t-tests comparing disability in individuals with and without missing values showed that those values were all missing completely at random (p = .14).

### Participants’ characteristics

Of all baseline characteristics, only pain catastrophizing was different between workers with a history of LBP and healthy controls (t_116_ = 2.82, p = .006), which is to be expected from a sample of individuals with a history of pain [46]. Baseline characteristics of all participants are reported in Table 2.

**Table 2:** Baseline characteristics of all participants. Comparisons are provided between individuals with LBP still enrolled at six months and all individuals with LBP. Baseline characteristics of all individuals with LBP are also compared to those of individuals without LBP.

	Individuals with LBP still enrolled at 6 months	Comparison using t-tests	Individuals with LBP initially recruited for the study	Comparison using t-tests	Individuals without LBP
	Total (77)		Total (100)		Total (19)
	*Mean ± SD*	*t*	*Mean ± SD*	*t*	*Mean ± SD*
	*[min-max]*	*p*	*[min-max]*	*p*	*[min-max]*
**Age (years)**	37.0 ± 11.0	t = 0.26	36.6 ± 12.1	t = 1.69	31.5 ± 11.0
	[19–59]	p = .79	[19–59]	p = .09	[20–58]
**Weight (kg)**	75.5 ± 18.6	t = 0.34	76.5 ± 18.7	t = 0.63	73.5 ± 11.6
	[45.4–150]	p = .73	[45.4–150]	p = .53	[58.0–95.0]
**Height (cm)**	170.6 ± 9.4	t = 0.16	170.9 ± 9.3	t = 0.92	173.1 ± 9.4
	[154.9–192]	p = .87	[154.9–192]	p = .36	[155.0–190]
**Disability (Roland-Morris /24)**	1.7 ± 2.5	**t = 3.30**	2.8 ± 2.6		NA
	[0–10]	**p < .001**	[0–11]		
**Mean clinical pain (VAS /100)**	21.3 ± 14.6	t = 0.40	22.6 ± 16.3		NA
	[3–65]	p = .39	[1–75]		
**Pain catastrophizing scale (PCS /52)**	13.2 ± 11.0	t = 0.17	13.5 ± 10.9	**t = 2.82**	5.8 ± 7.3
	[0–52]	p = .87	[0–52]	**p = .006**	[0–27]
**Pain vigilance**	35.5 ± 11.9	t = 0.00	35.5 ± 11.6	t = 1.29	31.4 ± 14.3
**(PVAQ/80)**	[2–59]	p = 1.00	[5–64]	p = .20	[2–58]
**Work satisfaction (MSQ/100)**	80.7 ± 11.4	t = 0.16	80.4 ± 10.9	t = 0.75	78.2 ± 11.9
	[53–100]	p = .88	[53–100]	p = .46	[46–99]
**Lumbar pain threshold (°C)**	45.0 ± 2.3	t = 0.40	44.8 ± 2.3	t = 0.18	44.7 ± 1.5
	[40.0–50.0]	p = .69	[38.5–50.0]	p = .86	[42.0–47.0]
**Lumbar pain tolerance (°C)**	50.1 ± 1.7	t = 0.29	49.9 ± 1.7	t = 1.79	49.2 ± 2.2
	[44.4–52.0]	p = .77	[44.4–52.0]	p = .08	[45.0–52.0]

### Pain thresholds and pain tolerances

Mean pain threshold and pain tolerance are reported in Table 3. There was no difference between controls and individuals with LBP with regards to pain threshold (low back: t_116_ = 0.176, p = .86 and forearm: t_116_ = 0.44, p = .66) or pain tolerance (low back: t_117_ = 1.79, p = .08 and forearm: t_117_ = 0.91, p = .37).

**Table 3:** Mean temperatures for pain thresholds and pain tolerances at each stimulation site (lower back and forearm) for healthy controls and individuals with a history of LBP.

	Pain thresholds		Pain tolerance	
	Lower back	Forearm	Lower back	Forearm
	*Mean ± SD*	*Mean ± SD*	*Mean ± SD*	*Mean ± SD*
	*[min-max]*	*[min-max]*	*[min-max]*	*[min-max]*
Healthy controls	44.7 ± 1.5	44.6 ± 1.6	49.2 ± 2.2	49.1 ± 1.8
	[42.0–47.0]	[42.0–47.0]	[45.0–52.0]	[44.5–52.0]
Individuals with a history of LBP	44.8 ± 2.3	44.4 ± 1.9	50.0 ± 1.7	49.4 ± 1.4
	[38.5–50.0]	[38.5–48.0]	[44.4–52.0]	[44.5–52.0]

### Pain ratings during HNCS

Mean pain ratings for each group before, during and after HNCS are reported in Table 4. HNCS significantly decreased cutaneous heat pain (F_2,230_ = 19.0, p < 0.001 η_p_^2^ = 0.14) in both groups. There was no interaction effect as both the patients and the control group showed a reduction in mean pain ratings during counter-stimulation. When combining data from both groups (main effect of conditions), planned contrasts revealed a significant reduction during HNCS (p < 0.001), but also a significant increase during the recovery period (p = .001).

**Table 4:** Mean pain ratings for cutaneous noxious heat applied to the lower back before (baseline), during (HNCS) and after (recovery) immersion of the left hand in cold water.

	Baseline	HNCS*	Recovery^†^
	*Mean ± SD*	*Mean ± SD*	*Mean ± SD*
	*[min-max]*	*[min-max]*	*[min-max]*
Healthy controls ^a^	39.1 ± 7.8	32.2 ± 16.5	43.8 ± 13.8
	[26–51]	[3–58]	[14–65]
Individuals with a history of LBP ^b^	37.9 ± 13.7	33.6 ± 19.4	42.5 ± 21.6
	[0–67]	[0–78]	[0–80]

^a^: 10 healthy controls reported an increase in pain perception during HNCS, whereas 9 reported a decrease in pain perception

^b^: 33 individuals with a history of LBP reported an increase in pain perception during HNCS, 5 reported no change and 62 reported a decrease in pain perception.

* Planned contrasts revealed a decrease (both groups) in pain perceptions during HNCS (p < .001)

† Planned contrasts revealed an increase (both groups) in pain perceptions during recovery (p = .001)

### Effects of experimental LBP on EMG and kinematics

ANOVAs revealed that cutaneous heat pain in the lower back did not significantly alter normalized RMS EMG during flexion, full flexion and extension phases (all p > 0.2 for the L4-L5 level and all p > 0.3 for the L2-L3 level) and on total trunk flexion angle (p = .5) for either group.

### Association between initial factors and disability at the six-month assessment

Of all variables entered in the correlation analysis, only the component pertaining to initial psychological symptoms (τ = 0.30, p < .001) and pain inhibition processes (r = -0.24, p = .038) were associated with disability observed six months after the initial assessment. Each of the psychological factor included in the principal component was also associated with disability six months later (PCS: τ = 0.34, p < .001; Hypervigilance: τ = 0.25, p = .007; fear-avoidance beliefs τ = 0.24, p = .009). No other variable was associated with disability observed at six months, including the initial total trunk flexion angle (τ = 0.02, p = .9), the components for initial pain thresholds and tolerance (τ = -0.13, p = .15), the initial neuromuscular adaptations (τ = 0.006, p = .96) and the initial neuromuscular responses to experimental pain (τ = 0.13, p = .23).

The first regression model (including the PCA for psychological symptoms and pain inhibition processes) showed that only psychological symptoms (β_clinical pain levels_ = .305, p = .009) contributed unique variance to disability at six months (R = 0.305, R^2^ = 0.093, F(1, 71) = 7, 16, p = 0.009). In the second model, the first step of the hierarchical regression including clinical pain at six months explained 21.3% of variance in disability (R = .462, R^2^ = 0.213, F(1, 64) = 17.366, p < .001). The second step of the regression showed that only pain inhibition contributed unique variance to disability at six months after controlling for pain levels. Together, clinical pain at six months (β_clinical pain levels_ = .484, p = .000) and initial pain inhibition processes (β_pain inhibition_ = -.307, p = .005) explained 30.7% of variance in disability at six months in individuals with chronic LBP (R = .554, R^2^ = 0.307, F(1, 63) = 25.92, p < .001). Tolerances for both independent predictors were high with .995 each indicating virtually no collinearity between current clinical pain levels and initial pain inhibition processes.

## Discussion

The main objective of this study was to determine, for a group of working individuals with a history of LBP, if psychological factors, neuromechanical variables, pain thresholds and pain tolerance thresholds, as well as the potency of pain inhibition processes were linked with disability six months later. Among these factors, the magnitude of pain inhibition was the only factor associated with disability after accounting for pain levels of individuals six months later. Besides, no association was observed for psychological factors, neuromuscular adaptations, neuromuscular responses to experimental pain, total trunk flexion angle, as well as pain thresholds and pain tolerance thresholds.

### Pain inhibition processes and disability

In the present sample of workers with a history of LBP, significant HNCS hypoalgesia was observed, and the results did not differ from those of the control group. These results are similar to what has already been observed in a previous study using a different protocol to assess pain modulation mechanisms [47]. Even though both groups were similar in terms of mean pain inhibition efficiency, there was a large amount of variability (see Table 3 for details), which has also been observed in studies focusing on patients with LBP [25] or other chronic pain conditions [48]. Nevertheless, the link between less potent pain inhibition and elevated disability six months later suggests a possible contribution of these mechanisms to the course of disability in individuals with LBP. As such, our results are consistent with the predictive value of conditioned pain modulation for the development of chronic pain [49] and for the efficacy of duloxetine [50], a drug that is thought to increase descending pain inhibition, a mechanism that contributes to HNCS hypoalgesia through DNIC-like effects (diffuse noxious inhibitory controls) [51].

In accordance with the current literature, and as mentioned above, altered pain inhibition processes may not be observed in all individuals reporting past or present LBP. Nevertheless, individuals with less potent pain inhibition processes could be vulnerable to develop more severe disability. The mechanism underlying this association, however, remains to be determined.

### Psychological symptoms and disability

Even though psychological symptoms reported by workers with a history of LBP were associated with disability six months later, their individual contribution vanished once entered in a model combining pain inhibition processes and clinical pain. These results add to those obtained by Scholich et al., 2012 who showed that clinical pain intensity can mask the contribution of otherwise meaningful variables to disability in individuals with low back pain [10]. After reviewing results of the correlation analyses, initial pain inhibition processes and initial psychological symptoms were inversely correlated suggesting that less potent pain inhibition processes are linked with increased psychological symptoms [52].

### Neuromuscular adaptations, movement patterns and disability

Changes in trunk muscle activation in individuals with LBP have been reported extensively in the literature [20] and are associated with punctual disability [27, 53]. Moreover, neuromuscular responses to experimental LBP were associated with disability in a cross-sectional study on LBP [27]. The current results show that EMG activity of LES, changes in EMG activity of LES induced by experimental LBP and total trunk flexion angle were not associated with disability six months later in the present sample. Previous studies have shown that neuromuscular adaptations in individuals with LBP persist beyond the experience of pain [21, 54] and as such, the lack of association between these measures and disability six months later is puzzling. One possible explanation comes from a recent study that showed neuromuscular adaptations in trunk muscle to be highly variable across individuals (each individual recruits a unique combination of trunk muscles to deal with pain) [55]. Therefore, the lack of association between neuromuscular adaptations and disability could be a product of high variability in acquired adaptations across individuals with a history of LBP. This phenomenon could also explain why some adaptations such as altered myoelectric silence during full flexion (the flexion relaxation phenomenon) [44] is present in a number of patients, whereas a normal EMG pattern is still observed in others [42, 53].

### Pain thresholds and disability

Previous studies exploring pain sensitivity in individuals with LBP reported localized and diffused hyperalgesia [22, 23], but a recent review of the literature argued against its possible implication in persistent or recurrent LBP [56]. Indeed, a number of studies reported no difference between pain sensitivity in patients with LBP compared to healthy controls [57, 58] and the current results corroborate these outcomes. This suggests that individual differences in pain thresholds and pain tolerance to cutaneous heat in the lower back and the upper limb are not linked with disability in workers with a history of LBP. These results support recent findings by LeResche et al., who reported that quantitative sensory testing measures were poor predictors of back pain four months after their assessment [59].

### Clinical implications

Based on the association between pain inhibition and disability six months later, the present results suggest that therapies aiming at enhancing pain inhibition processes can potentially be useful to reduce disability in individuals with a history of LBP. For instance, serotonin-norepinephrine reuptake inhibitors (SNRI) have been successfully used in the management of pain in chronic or recurrent LBP [60] and have shown to decrease disability [61]. SNRIs could act on restoring or improving pain inhibition [62, 63] and alleviating depressive symptoms [60], which are known to be linked to chronic back pain [64]. Even if pain inhibition processes only explained 9.4% of variance in future disability, the use of SNRIs could be considered in multidisciplinary approaches as SNRIs not only act on pain inhibition processes, but also help with depressive symptoms often observed in individuals with LBP [60].

### Limitations

The current design (longitudinal study) allowed for many characteristics to be evaluated as possible predictors of future disability in individuals with past or present LBP. Still, some reservations regarding the current results might emerged as a consequence of the attrition of some participants (from 100 at the initial evaluation to 77 at the second). As mentioned earlier, participants who dropped out of the study were no different than those who stayed. Moreover, no distinct pattern emerged in missing values amongst participants who remained enrolled in the study. It could be argued that the relatively low disability observed in the current sample prevents generalization of the results to moderate or severe LBP. This limitation is inherent to the choice of the sample characteristics, considering that we were interested in individuals with a history of LBP who could still work despite functional limitations and whose work performance was potentially reduced by these limitations [65]. Therefore, results may not be generalizable to all individuals with LBP, but they apply to workers with LBP, a population that is not often specifically investigated.

## Conclusion

The presentation of chronic low back pain is often characterized by recurring symptoms, suffering and disability, rather than by objective tissue abnormalities. Even if numerous factors have been associated with the condition, only pain inhibition processes were linked to disability six months later after controlling for clinical pain levels. Even though the optimal strategy to prevent future disability in individuals with a history of LBP remains to be determined, the use of interventions aimed at restoring pain inhibition processes combined with a reduction of pain through punctual analgesic interventions could prove helpful to limit future disability in these individuals.

## Supporting Information

S1 Datasets Used for all analyses. Includes a file for EMG data and a file for all other variables.
(ZIP)
